# The *Arabidopsis* Plant Intracellular Ras-group LRR (*PIRL*) Family and the Value of Reverse Genetic Analysis for Identifying Genes that Function in Gametophyte Development

**DOI:** 10.3390/plants2030507

**Published:** 2013-08-09

**Authors:** Nancy R. Forsthoefel, Kendra A. Klag, Barbara P. Simeles, Rachel Reiter, Lauren Brougham, Daniel M. Vernon

**Affiliations:** Biology Department, Whitman College, Walla Walla, WA 99362, USA

**Keywords:** *Arabidopsis*, gametophyte, knockout mutant, leucine-rich repeat, pollen, reverse-genetics

## Abstract

*Arabidopsis thaliana* has proven a powerful system for developmental genetics, but identification of gametophytic genes with developmental mutants can be complicated by factors such as gametophyte-lethality, functional redundancy, or poor penetrance. These issues are exemplified by the Plant Intracellular Ras-group LRR (*PIRL*) genes, a family of nine genes encoding a class of leucine-rich repeat proteins structurally related to animal and fungal LRR proteins involved in developmental signaling. Previous analysis of T-DNA insertion mutants showed that two of these genes, *PIRL1* and *PIRL9*, have an essential function in pollen formation but are functionally redundant. Here, we present evidence implicating three more *PIRL*s in gametophyte development. Scanning electron microscopy revealed that disruption of either *PIRL2* or *PIRL3* results in a low frequency of pollen morphological abnormalities. In addition, molecular analysis of putative *pirl6* insertion mutants indicated that knockout alleles of this gene are not represented in current *Arabidopsis* mutant populations, suggesting gametophyte lethality may hinder mutant recovery. Consistent with this, available microarray and RNA-seq data have documented strongest *PIRL6* expression in developing pollen. Taken together, these results now implicate five *PIRL*s in gametophyte development. Systematic reverse genetic analysis of this novel LRR family has therefore identified gametophytically active genes that otherwise would likely be missed by forward genetic screens.

## 1. Introduction

The proper development of gametophytes is critical for plant reproduction. An integrative understanding of gametophyte formation and function requires identification of genes active in the haploid phase and elucidation of their roles and functional relationships. With a complete and well-annotated genome sequence and well-established genetic resources, *Arabidopsis thaliana* has proven a valuable model system for identifying genes with functions in gametophyte development.

Forward genetic approaches have proven effective for identifying loci with gametophytic functions [1,2,3,4,5]. One strategy for forward genetic screens has been to look for distorted segregation of mutagen-encoded marker phenotypes, such as T-DNA encoded antibiotic resistance, to identify plants with reduced transmission of marker phenotypes [6,7]. Forward genetic screens have also targeted mutants exhibiting abnormal gametophtye development or altered expression of gametophyte-specific reporter constructs [8,9,10,11,12]. Forward screens have been especially successful in pollen, leading to identification of mutants with specific cellular defects in male gametophyte development (e.g., [13,14]). Taking into account these studies and others, Muralla *et al*. [15] compiled a dataset of 173 loci genetically confirmed to be essential for gametophyte development. Complementing genetic efforts, transcriptome studies have successfully cataloged many gametophytically expressed loci, particularly in pollen, providing a starting point for further functional analyses and a resource for confirming gametophytic expression of genes identified by other methods [16,17,18,19].

A number of factors can complicate identification of gametophyte genes by forward genetics. Some are issues that can hinder any genetic screen. One of these is functional redundancy among gene family members, which can interfere with the expression of a mutant phenotype in homozygous mutants defective in just one locus [20,21,22]. Another factor is incomplete penetrance or poor expressivity of mutant alleles, which can result in subtle or low-frequency defects that can potentially be missed in large-scale genetic screens. These factors can be related because partial redundancy can be one potential cause for incomplete penetrance or variable expressivity; however this need not always be the case, as non-essential genes or genes needed only under certain environmental conditions may also exhibit subtle or variable phenotypes.

With the popularity of insertion mutagenesis in model systems such as *Arabidopsis*, another factor that complicates gametophyte developmental genetics is the haploid nature of the gametophyte phase, which can prevent recovery of knockout mutant alleles of genes essential in both the male and female gametophyte [4]. Gametophytic developmental genes account for only about 8% of documented *Arabidopsis* loss-of-function phenotypes [22], and thus may be underrepresented in mutant collections [5,23,24]. Indeed, despite the generation of over 325,000 sequence-tagged *Arabidopsis* insertion mutant lines, insertion alleles have been persistently elusive for approximately 12% of loci [25]. It is likely that at least a portion of these are gametophyte-essential genes for which *bona fide* knockout alleles cannot be recovered. However, the exact numbers of such genes is difficult to determine because of their very nature-mutant lethality stands as a barrier to identification by insertion mutation [4,23]. What is clear is that, despite the successful genetic identification of numerous gametophytic developmental genes in recent years, others are likely to go undetected in many mutant screens, due to the various factors described above.

The *Arabidopsis* *PIRL1* and *PIRL9* genes are a case in point. *PIRL*s encode a plant-specific class of leucine-rich repeat proteins related to Ras-interacting LRRs that take part in developmental signaling in animals and fungi [26]. PIRLs are distinct from larger, well-characterized classes of plant LRR proteins such as NBS-LRR pathogen response proteins [27] or LRR-receptor-like kinases, many of which have well-established functions in development [28,29,30]. Characterization of *Arabidopsis* T-DNA insertion mutants demonstrated that *PIRL1* and *PIRL9* have an essential role early in pollen development, but are functionally redundant. Plants homozygous for single mutations in either gene do not display obvious defects in pollen development or transmission, but *pirl1*;*pirl9* double mutant microspores undergo consistent developmental arrest and are inviable [31,32]. Thus, detailed reverse genetic analysis revealed a functional context for these two genes whose gametophyte phenotypes would otherwise have remained masked by functional redundancy.

Here, we provide evidence implicating three additional members of this novel LRR family in gametophyte development. We have examined pollen produced by plants homozygous for T-DNA knockout mutations in *PIRL2* and *PIRL3,* and observed a low frequency of abnormal pollen in *pirl2* and *pirl3* mutants, revealing that these genes act in pollen development, but by themselves do not likely have a large enough effect to be detected in forward genetic screens. Furthermore, analysis of another locus, *PIRL6*, provided two lines of indirect evidence that this gene may function in gametophytes: (1) transcripts are detected primarily in gametophtyes and reproductive tissues, especially pollen; and (2) plants homozygous for putative knockout alleles still express *PIRL6* mRNA, indicating that they are not *bona fide* knockout mutants and suggesting that gametophyte lethality may be preventing recovery of null mutant alleles. Taken together with prior analysis of *PIRL1* and *PIRL9*, these finding now suggest that five of the nine *Arabidopsis* *PIRL*s may be involved in gametophyte development.

## 2. Results and Discussion

### 2.1. The Arabidopsis PIRL Family and Insertion Mutants

The *PIRL*s were originally recognized in the completed *Arabidopsis* genome sequence as encoding a distinct plant-specific class of LRRs related to animal and fungal Ras-group LRRs [26]. Homologs have since been identified in rice [33]. Based on sequence similarity and intron/exon positions, the nine *Arabidopsis* *PIRL*s group into three subfamilies, with *PIRL1*, *PIRL2*, *PIRL3*, and *PIRL9* constituting the largest [26]. Candidate T-DNA insertion alleles have been identified for all members of the gene family by PCR screening of original Wisconsin mutant pools [34] or by mining publically available sequence-tagged insertion mutant collections [35,36,37,38]. Allele and polymorphism designations and sources for mutants used in this study are listed in Table 1. With one exception (*pirl6-2*), inserts were located within transcription units. The *pirl1* and *pirl9* alleles were previously confirmed to be true knockouts by RT-PCR [31]. *pirl2*, *pirl3* and candidate *pirl6* mutants are described below in Section 2.3 and Section 2.4. 

**Table 1:** Plant Intracellular Ras-group LRR (PIRL) genes and mutant alleles included in this study.

*PIRL* *(sub-family)*	AGI Locus	Mutant alleles in this study	Insert position (nucleotide position)
*PIRL1 (I)*	At5g05850	*pirl1-1 ^a^*	Intron I (+1107)
*PIRL9 (I)*	At3g11330	*pirl9-1 ^a^*	Exon I (+357)
*PIRL2* *(I)*	At3g26500	*pirl2-1 ^a^*	Exon I (+526)
		*pirl2-2* [SALK_138743] ^b^	Exon III (+1147)
*PIRL3* *(I)*	At1g12970	*pirl3-1 ^a ^*	Exon III (+1640)
		*pirl3-2* [SALK_033703] ^b^	Exon I (+514)
*PIRL*6 *(II)*	At2g19330	*pirl6-1* [SAIL574A05] ^c^;	Exon I (+182)
		*pirl6-2* [WISCDSLOX393-396L14] ^c^	Promoter region (-272)

^a^ Mutant isolated from Wisconsin T-DNA populations [34] by PCR screening as described in Forsthoefel *et al*. (2005); ^b^ Mutant line from Salk T-DNA mutant collection [35]; see [39] for visual maps; ^c^ Mutant lines from SAIL or WiscDsLox T-DNA mutant collections ([36,37]; see [39] for visual maps.

### 2.2. PIRL1 and PIRL9 Illustrate Functional Redundancy in Pollen

**Figure 1:**
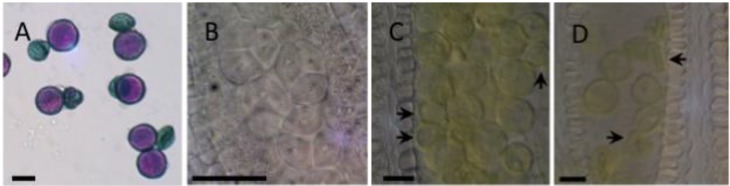
Developmental arrest in *pirl1*, *pirl9* pollen. (**A**) Alexander-stained dehisced pollen from a *pirl1/PIRL1*, *pirl9* plant, segregating at 50% for the *pirl1*, *pirl9* mutant phenotype. Purple stain indicates viable pollen; (**B**) Nomarski DIC microscopy of post-meiotic tetrads in a *pirl1/PIRL1*, *pirl9* anther in which double mutant microspores are segregating but cannot be clearly identified; (**C**) A *pirl1/PIRL1*, *pirl9* anther with mitotic stage pollen; arrows indicate developmentally arrested pollen; (**D**) A mature *pirl1/PIRL1*, *pirl9* anther; arrows indicate shrunken inviable double mutants segregating within the pollen population. Scale bars = 20 µm

Disruption of both *PIRL1* and *PIRL9* together results in pollen lethality [31]. Figure 1 illustrates developmental arrest in pollen produced by *pirl1/PIRL1*;*pirl9* plants, which segregates 50% for the double mutant genotype. Mature pollen from dehisced anthers was approximately 50% inviable based on Alexander’s staining, in which inviable grains appear shrunken and blue-green (Figure 1A). Nomarski DIC microscopy of developing anthers showed that segregating *pirl1*;*pirl9* microspores were not distinguishable in tetrads just after meiosis (Figure 1B), but became clearly evident later, first appearing in mitotic phase anthers as smaller, arrested grains and later appearing shrunken and inviable (Figure 1C,D). These observations are consistent with prior genetic and fluorescence microscopy results that demonstrated that *PIRL1* and *PIRL9* act post-meiosis to affect pollen formation [31,32].

### 2.3. PIRL2 and PIRL3 Affect Pollen Morphology

**Figure 2:**
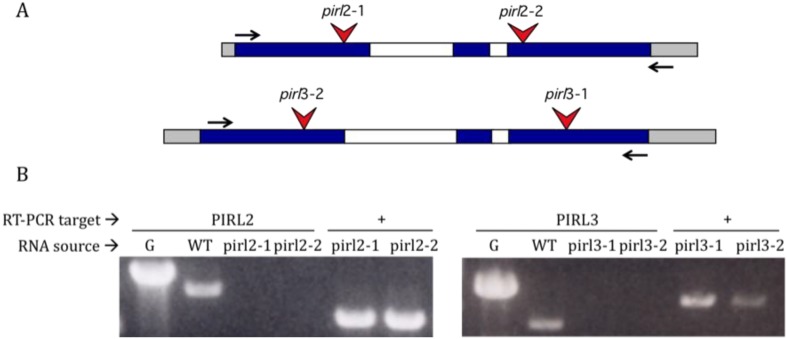
*PIRL2* and *PIRL3* T-DNA insertion alleles and knockout status. (**A**) *PIRL2* and *PIRL3 gene maps*, with exons shaded and introns shown in white; light gray shading indicates UTR regions. Positions of T-DNA insertions in *pirl2-1*, *pirl2-2*, *pirl3-1*, *pirl3-2* alleles are indicated by red arrows. Horizontal arrows indicate positions of RT-PCR primers used for part B; (**B**) Confirmation of knockout status in *pirl2* and *pirl3* DNA insertion lines. RT-PCR was carried out with gene-specific primers on total RNA isolated from flowers or leaves of homozygous mutant *(pirl2-1*, *pirl2-2*, *pirl3-1*, or *pirl3-2*, as indicated) or wild-type (WT) controls. G, *PIRL* gene (with introns) amplified from genomic DNA.(+) lanes contain RT-PCR products for *PIRL9* or *PIRL1* amplified from *pirl2* or *pirl3* mutants, respectively, as positive controls for RNA quality and RT-PCR efficacy.

To determine if other members of *PIRL* sub-family I are involved in pollen development, we identified two independent T-DNA insertion mutants for each of *PIRL2* and *PIRL3* (Figure 2). T-DNA inserts in the *pirl2*-*1*, *pirl2*-*2*, *pirl3*-*1* and *pirl3*-*2* alleles were located within exon sequences at positions upstream of or within the region encoding the highly conserved *PIRL* LRR domain (Figure 2A; see Table 1 for nucleotide positions). The knockout status for all of these lines was verified by RT-PCR on homozygous mutants, using gene specific primers that targeted the full-length *PIRL2* or *PIRL3* ORFs (Figure 2B). The exon positions of the *pirl2* and *pirl3* T-DNA inserts make it highly unlikely these alleles give rise to undetected truncated mRNAs with any residual functional potential, especially given their disruption of the conserved LRR domain, the most prominent feature of the *PIRL* gene products [26]. 

**Figure 3:**
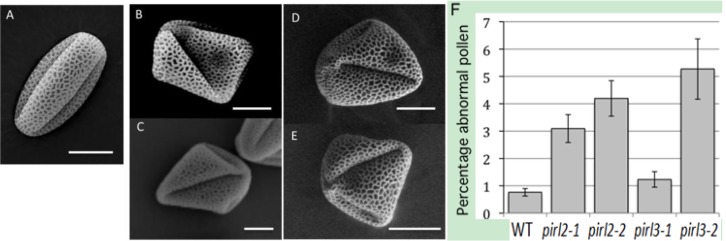
Mutations in *pirl2* or *pirl3* result in abnormal pollen. Dehisced pollen produced by wild-type *Arabidopsis* (**A**), or by *pirl2* (**B**,**C**) or *pirl3* (**D**,**E**) homozygotes was viewed by scanning electron microscopy. Scale bars, 10 µm. (**F**) Percentages of abnormal pollen estimated by light microscopy. Values are means derived from pollen obtained from four to nine plants of each genotype; standard errors are shown. Mean values and total sample sizes: Wild type (WT), 0.76% (n = 2826); *pirl2*-*1*, 3.09% (n = 4913); *pirl2*-*2*, 4.20% (n = 2872); *pirl3*-*1*, 1.23% (n = 3588); *pirl 3*-*2*, 5.27% (n = 1441).

We examined pollen produced by *pirl2* and *pirl3* mutant homozygotes. Dehisced pollen were collected and subjected to scanning electron microscopy (SEM). Abnormal pollen was observed both in *pirl2* and in *pirl3* mutant lines (Figure 3). Abnormal pollen varied from shrunken to large, irregular, and angular in shape. This contrasts to what was observed for *pirl1*;*pirl9* pollen, which were consistently severely shrunken and inviable (Figure 1A and [31]). Penetrance was determined by light microscopy of Alexander-stained pollen from multiple plants from each mutant line and from wild-type controls. The *pirl2* and *pirl3* phenotypes were poorly penetrant but were reproducibly and consistently observed for both mutant alleles of each gene (Figure 3F). The penetrance values in Figure 3 are conservative estimates because they were derived using light microscopy, which cannot detect modest morphological defects due to the fact that pollen are hydrated and viewed at much lower magnification than with SEM. However, this method had the advantage of allowing for much larger sample sizes than did SEM. 

Other features of the *PIRL2* and *PIRL3* genes are consistent with a role in pollen. While both genes are broadly expressed throughout plant development, based on RT-PCR [26], available microarray and RNA-seq data show *PIRL3* expression is at its highest level in pollen [19,40,41]. Microarray data for *PIRL2* are not available because it is unfortunately among the loci not represented on most *Arabidopsis* microarrays. Recent RNA-seq data suggest a very low level of *PIRL2* expression in dehisced pollen, however earlier stages of pollen development that would be the most likely to affect pollen morphology were not tested [19]; major changes in the pollen transcriptome occur after pollen morphogenesis as pollen prepare for dehiscence and later germination [17]. Notably, based on sequence, *PIRL2* appears to be an ortholog of SF17, a sunflower gene expressed predominantly in pollen [42].

The morphological defects observed in *pirl2* and *pirl3* pollen strongly suggest that these genes act after meiosis, in microspores or pollen. This is based on their relatively robust appearance in comparison to *pirl1*, *pirl9* microspores, which also act after meiosis but arrest just prior to pollen mitosis I. Hypothetically, crossing *pirl2* and *pirl3* into a *qrt1* background could confirm post-meiotic activity of *pirl2* and *pirl3* [3]; however, because of the low penetrance, phenotype segregation within *qrt1* tetrads would not provide meaningful results, as tetrads would rarely feature more than one defective pollen grain.

While the *pirl2* and *pirl3* mutations affect pollen they apparently do not disrupt development sufficiently to affect efficacy of transmission. Therefore, these genes would not be classified as essential [4], and their involvement in pollen development would not likely be detectable in forward-genetic screens relying on segregation distortion. We considered the possibility that *pirl2* and *pirl3* phenotypes might be largely masked by redundancy in this gene pair, as was the case for *pirl1* and *pirl9* [31]. However, this does not appear to be the case, as Alexander’s staining of *pirl2*;*pirl3* double mutant pollen indicated a phenotype frequency no more severe than that observed for the single mutants. 

### 2.4. Analysis of Putative pirl6 Knockout Mutants and PIRL6 mRNA Expression.

**Figure 4:**
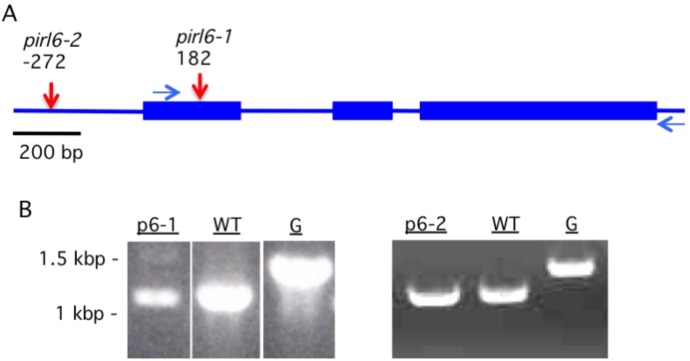
*PIRL6* gene structure, T-DNA insertion alleles, and knockout status. (**A**) The *PIRL6* locus (At2g19330), with exons shown as blue rectangles, introns and flanking regions as lines. Positions of T-DNA insertions in putative knockout mutants *pirl6-1* (SAIL574A05) and *pirl6-2* (WISCDSLOX393-396L14) are indicated by arrows. Horizontal arrows indicate positions of RT-PCR primers used for part B; (**B**) Detection of *PIRL6* transcripts in *pirl6-1* and *pirl6-2* putative T-DNA knockout lines. RT-PCR was carried out with *PIRL6*-specific primers on total RNA isolated from flowers of homozygous mutant (p6-) or wild-type (WT) plants. G, *PIRL6* gene (with introns) amplified from genomic DNA. RT-PCR specificity was confirmed by sequencing of gel purified reaction products.

We extended reverse genetic analysis to *PIRL* subfamily II by identifying and characterizing putative knockout mutations in *PIRL6*. We identified only two candidate insertion mutations in this locus, which we labeled *pirl6*-*1* (SAIL574A05) and *pirl6*-*2* (WISCDSLOX393-396L14). Analysis of junction sites confirmed the position of the insertions in the first exon and the presumed promoter region, respectively (Figure 4A). However, RT-PCR of RNA from flowers of mutant homozygotes clearly indicated the expression of intact *PIRL6* transcripts, demonstrating that neither allele was a null mutation (Figure 4B). In the case of *pirl6-2*, the residual gene expression may be explained by the position of the insertion outside the transcription unit. The exact reason for *PIRL6* transcript expression in *pirl6-1* cannot easily be determined, but it is likely that this line contains some type of T-DNA associated rearrangement or duplication, such that an intact copy of the locus remains, possibly at a different chromosomal location. Such duplications and rearrangements are not uncommon in T-DNA mutagenized populations [25,43,44].

The presence of only two candidate *pirl6* knockout alleles, and the finding that neither is really a *bona fide* knockout, suggest that the gene could be gametophyte-essential. We acknowledge that this is negative evidence, and we cannot rule out that the dearth of *pirl6* candidate alleles is just due to chance. For example, the gene could lie in a chromosomal region not prone to Agrobacterial T-DNA insertion. However, we investigated the neighboring locus, At2g19340, which lies immediately adjacent to *PIRL6*, and identified six candidate insertion lines [39]. Thus, this region of chromosome 2 is readily accessible to T-DNA insertion mutagenesis. It therefore appears that some other feature of *PIRL6*, such as an essential gametophyte function, may be the reason knockout alleles have not been generated.

**Figure 5:**
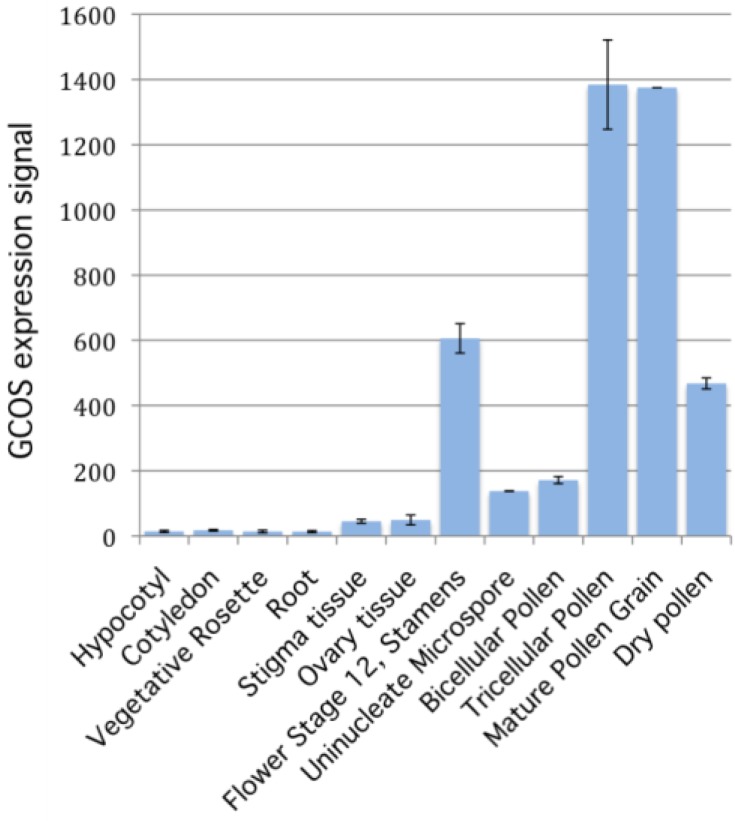
Detection of *PIRL6* transcripts in reproductive tissues, gametophytes, and representative vegetative organs. Data originated from multiple published microarray experiments (see Table 2) and was accessed via the *Arabidopsis* eFP browser [41].

To determine if *PIRL6* expression was consistent with a gametophyte function, we investigated *PIRL6* transcript levels in existing microarray databases and a pollen RNA-seq dataset. Our aim was not to determine whether PIRL6 expression is gametophyte specific, because essential genes need not exhibit expression at one specific location or developmental stage. Rather, we sought confirmation that *PIRL6* was expressed in male and female gametophytes—a prerequisite for gametophytic function. Multiple transcriptome studies accessed via both the eFP browser [41] and Genevestigator [45,46] suggested that *PIRL6* transcripts are indeed expressed primarily in male and female reproductive structures and gametophytes (Figure 5; Table 2). Expression levels were very low throughout vegetative development, but were high in microspores and developing pollen, strongly suggesting a function in the male gametophyte. These microarray findings were recently confirmed by a large-scale RNA-seq study comparing mature pollen and seedling transcriptomes [19]. Female reproductive organs also expressed *PIRL6* above background levels seen in vegetative tissues, although expression was much lower than in pollen. Taken together with the negative evidence from the knockout analysis described above, these results are consistent with a gametophytic role for *PIRL6.* Direct genetic evidence for *PIRL6* gametophyte function will require targeted gametophyte-specific gene knockdown in transgenic plants, tilling for non-lethal point mutations, or fortuitous identification of less severe mutant alleles in a forward genetic screen.

**Table 2:** Multiple microarray studies as well as RNA-seq document *PIRL6* expression in gametophytes and reproductive organs.

	Expression database or dataset	Primary reference
**Microspores, pollen, stamens**	eFP browser, developmental map [41]	[47]
	eFP browser, tissue specific map	[17]
	Genevestigator [46]	[16]
	Genevestigator	[48]
	Pollen RNA-seq dataset	[19]
**Ovaries, ovules, carpels**	Genevestigator	[49]
	eFP browser, developmental map	[47]
	eFP browser, tissue specific map	[50]

## 3. Experimental

### 3.1. Mutant Identification and Confirmation of T-DNA Insert Position

Seeds for prospective mutant lines were obtained from the *Arabidopsis* stock center at Ohio State University. Plants were grown in climate-controlled growth chambers under a 16 h light/8 hours dark regime as previously described [44]. *pirl1-1*, *pirl9-1*, *pirl2-1*, and *pirl3-1* alleles were identified by manual PCR screening of T-DNA mutagenized mutant pools from the Wisconsin collection [34], and insert position and segregation confirmed by PCR of T-DNA:plant DNA junction fragments as described by Forsthoefel *et al.* [26]. *pirl2-2* and *pirl3-2* were identified in the SALK mutant collection [35], and T-DNA insert positions and allele segregation were confirmed using the forward and reverse gene specific primers listed below in combination with T-DNA-specific primers specific for the SALK collection T-DNA border sequences [35]. All mutant lines were subjected to serial backcrossing to wild type prior to phenotype analysis. Candidate *pirl6* insertion mutants were identified as At2g19330 polymorphisms at TAIR [39]. In combination with insert-specific border primers appropriate for either the SAIL mutant collection [36] (primer LB3, for *pirl6-1*) or the WISCDSLOX collection [37] (primer p745, for *pirl6-2*), *PIRL6* locus-specific primers were used for genomic DNA analyses to assess the presence of inserts and identify homozygous individuals: forward (F), TAGTGAGAACAATCCTTTTGATTTGGTCA; reverse (R), AGAAATAGTCAAATA-GGGACCTGGTGCAA. In the absence of a *pirl6* phenotype and with residual gene activity in putative knockout lines, homozygotes lines were unambiguously identified by presence of the insert in 100% of F1 progeny from self-fertilized individuals.

Knockout status of putative homozygous mutants was determined by using RT-PCR on total flower RNA to detect the presence or lack of target mRNA. RNA preparations and RT-PCR reactions were carried out as described by Cushing *et al.* [51]. Gene specific forward and reverse primer combinations targeted full-length transcripts: *PIRL2* F, GCCGCCGTCGCCGCCGTCTAT GGCCGCGC; *PIRL2* R, CGGAGTTTGTTCAGCTGG; *PIRL3* F, CTCTCCTACGTCCTCCACCA; *PIRL3* R, GCTTCTTAGCTGCACCACCA; *PIRL6* F, ATGCGAGGAGGCATATCATC; *PIRL6* R, CGACGTGGAGAGAACATTCC. *PIRL* gene sequences are sufficiently divergent to allow design of gene-specific primers without cross-reactivity to other gene family members [26]. Specificity for all RT-PCR primer pairs was verified by prior sequencing of gel-purified RT-PCR reaction products. Products were isolated with the QIAquick gel extraction kit (Qiagen, Valencia, CA, USA) and sequenced by the by the University of Arizona DNA sequencing core facility (Tucson, AZ, USA).

### 3.2 Microscopy

Anthers of selected developmental stages [52] were microdissected from developing buds and immediately cleared on slides in Hoyer’s solution (100 g chloral hydrate, 5 mL glycerin, 7.5 g gum Arabic, 30 mL ddH_2_O) for 15 min. Cover slips were added and samples were viewed under an Olympus BX60 microscope equipped with Nomarski DIC optics. For light microscopy pollen were stained and viewed using the procedure described in Forsthoefel *et al*. [31]. For penetrance determination, samples were obtained from 4–9 plants of each genotype. The percentage of defective pollen was determined for each sample; mean percentages for each genotype were plotted along with standard error. Total N values are provided in Figure 3.

For scanning electron microscopy, dehisced pollen from plants homozygous for the indicated genotypes were transferred by lightly touching flowers onto carbon tape adhered to a sample stub. Stubs were air-dried for 24–72 hours and either viewed directly under high vacuum conditions at 1 KV, or gold coated with a Cressington 108 sputter coater for 20 seconds and viewed under high vacuum settings at 12.5–15 KV, using an FEI Quanta200 scanning electron microscope.

## 4. Conclusions

Systematic reverse genetic analysis has now implicated five of the nine *Arabidopsis* *PIRLs* in gametophyte development. For differing reasons, it is unlikely that any of these genes would have been implicated in gametophyte development by forward genetics. In the cases of *PIRL1* and *PIRL9*, essential pollen function was masked by almost complete redundancy, while for both *PIRL2* and *PIRL3*, loss of function under laboratory growth conditions does not generate a phenotype of adequate scope to be detected in a forward screen. In the case of *PIRL6*, we can offer only indirect evidence for a gametophytic role: molecular characterization of available insertion mutants reveals that *bona fide* knockouts are not present in current sequence-tagged *Arabidopsis* mutant populations, and flower specific mRNA expression and high pollen expression support a possible gametophyte function. Now that the biological contexts of these *PIRLs*’ functions have been defined, future work can focus on the cellular and biochemical roles of these novel, previously uncharacterized LRR genes, to better understand their contributions to plant reproductive development.

The value of reverse genetic strategies for identifying developmental genes were recognized by Berg *et al*. [23], who suggested that some genes with functions in reproductive development will escape identification, even as *Arabidopsis* mutagenesis efforts approach genome saturation. The results reported here support that point and underscore the value of detailed reverse genetics for achieving a full understanding of the genetic components of gametophyte formation and function.
